# Diverging effects of tumor necrosis factor inhibitors and conventional synthetic disease-modifying antirheumatic drugs on immunosenescence and inflammageing in rheumatoid arthritis: a cross-sectional analysis

**DOI:** 10.1186/s12979-025-00508-w

**Published:** 2025-05-22

**Authors:** Tobias Schwarz, Giovanni Almanzar, Sebastian Völkl, Martin Feuchtenberger, Johannes Leierer, Christian Schmidt, Frank Deininger, Hans-Peter Tony, Marc Schmalzing, Martina Prelog

**Affiliations:** 1 Department of Pediatrics, Pediatric Rheumatology and Special Immunology, University Hospital Würzburg, University of Würzburg, Würzburg, Germany; 2 Department of Internal Medicine II, Rheumatology/Clinical Immunology, University Hospital Würzburg, University of Würzburg, Würzburg, Germany; 3 MVZ MED|BAYERN OST, Burghausen, Germany; 4 Department of Internal Medicine IV (Nephrology and Hypertension), Medical University Innsbruck, Innsbruck, Austria; 5 Department of Haematology and Oncology, University of Greifswald, Greifswald, Germany; 6 Practice for Rheumatology, Haugerpfarrgasse 7, Würzburg, Germany

**Keywords:** Rheumatoid arthritis, Immunosenescence, Inflammageing, Tumor necrosis factor inhibitor, Conventional synthetic disease-modifying antirheumatic drug

## Abstract

**Background:**

Immunosenescence is characterized by a decline in naive T cells, a reduced T cell receptor repertoire, and the accumulation of terminally-differentiated and unspecifically-activated proinflammatory cells, a process called inflammageing. Premature immunosenescence is thought to be pathogenetically relevant in rheumatoid arthritis (RA), either by posing a risk factor for its development, or by advancing the rheumatic disease as a result of excess antigenic and inflammatory stimulation. We investigated parameters of immunosenescence in RA patients treated with conventional synthetic disease-modifying antirheumatic drugs (csDMARDs) only compared to patients treated additionally or exclusively with a tumor necrosis factor inhibitor (TNFi) and age-matched healthy controls to investigate the effect of RA treatment on age-associated T cell phenotypes and functions.

**Results:**

The csDMARD-only treated patients, compared to the TNFi-treated patients and healthy controls, displayed an enhanced age-dependent decline in CD31^+^ recent thymic emigrants (RTE) and Interleukin-7 (IL-7)-receptor α-chain (CD127)-expressing CD4^+^ T cells participating in IL-7-associated homeostatic proliferation, a diminished proliferation of RTE and CD127^+^ T cells, as well as reduced T cell receptor excision circle (TREC) counts. However, whereas the RA patients exhibited reduced proportions of unspecifically activated IFNγ- and IL-17-producing T cells, TNFi initiation induced an increase in these proinflammatory cells.

**Conclusions:**

Whereas a TNFi treatment seems to counteract the non-inflammatory aspects of immunosenescence, it induces increasing proportions of terminally-differentiated, cytokine-producing effector memory T cells, requiring awareness as possibly contributing to secondary autoimmune phenomena in RA.

**Supplementary Information:**

The online version contains supplementary material available at 10.1186/s12979-025-00508-w.

## Background

With advancing age of an individual, the immune system undergoes changes summarised under the term immunosenescence. The associated immunological alterations could be divided into non-inflammatory and inflammatory changes. Non-inflammatory hallmarks of immunosenescence are a decline in thymic function, accompanied by alterations in the peripheral T cell homeostasis [1], leading to a contraction of the T cell repertoire [2]. As a consequence of thymic involution, the number of CD31^+^ recent thymic emigrants (RTE) decreases [3], as does the frequency of T cell receptor (TCR) excision circle (TREC) containing naive T cells in the peripheral blood [4]. The loss of thymic productivity is compensated by a prolonged homeostatic maintenance of naive T cells in lymphoid tissue [5], a process requiring Interleukin-7 (IL-7) and TCR signalling, mediated by low-affinity self-peptide/major histocompatibility (MHC) recognition [6, 7]. Despite these compensatory mechanisms, T cell ageing manifests in a diminished ratio of naive to memory T cells [8], and an increasing CD4/CD8-ratio [9]. The increased post-thymic proliferation of naive T cells is accompanied by a contraction of the TCR repertoire with clonal expansion of certain TCRs [10, 11], a process that could lead to the selection of T cells with increased affinity to self- or neoantigens, thus increasing the risk for autoimmunity [12, 13].

Regarding the inflammatory characteristics of immunosenescence, in a process also called inflammageing, terminally differentiated and unspecifically activated proinflammatory cells accumulate [14], whereas the functional properties of regulatory T cells (Treg cells) subside [15].

Taken together, the ageing of the immune system results in a declining host protection against foreign pathogens and cancer cells, an increased risk for autoimmunity [12, 13, 16], vascular and neurodegenerative diseases [17], as well as a reduced response to vaccination [18].

One of the autoimmune diseases, in which premature or accelerated ageing is supposed to be pathogenetically relevant, is rheumatoid arthritis (RA) [19–25]. Based on genetic risk factors [26] and until now unknown environmental triggers, its immunopathogenesis starts with the production of autoantibodies against post-translationally modified proteins, often years before the first onset of symptoms [27]. An ongoing immune system remodelling [28] finally ends up in a loss of tissue tolerance and the onset of joint inflammation [29].

Some known molecular mechanisms contributing to an accelerated immune ageing in RA are a reduced expression of telomerase and the DNA damage sensing kinase ATM, as well as a decline in p53. This leads to an increased apoptotic loss of T cells with accumulation of terminally-differentiated cells that are resistant to apoptosis [30]. Furthermore, the disease associated enhanced loss of peripheral T cells induces homeostatic proliferation involving the cytokines IL-7 and IL-15. These homeostatic cytokines were shown to decrease the signalling threshold for TCR activation [31], thus rendering naive T cells responsive to low affinity self-antigens and thereby initiating differentiation to autoreactive memory T cells.

Although it is difficult to distinguish to which extend accelerated ageing drives the development of RA or whether it is a consequence of the chronic inflammatory process, it seems that ageing and inflammation form a self-perpetuating vicious circle, in which the premature ageing of the immune system advances the rheumatic disease and vice versa [32, 33]. Along that line, the above mentioned division of immunosenescence in inflammatory and non-inflammatory alterations can not be separated from one another, as inflammation itself affects the non-inflammatory part as well. Furthermore, immunosenescence itself seems to contribute to systemic ageing of the organism [34]. Therefore, the currently available anti-rheumatic treatments should not only be investigated regarding their effects on the local inflammatory processes, but also regarding their influence on the ageing of the immune system. To date, however, the effects of conventional synthetic disease-modifying anti-rheumatic drugs (csDMARDS) or biologic agents on the ageing-associated T cell phenotype and function have not yet been the subject of an exploratory analysis. Therefore, we compared parameters of immunosenescence between RA patients, treated with csDMARDs only, and patients, treated additionally or exclusively with tumor necrosis factor inhibitors (TNFi).

## Methods

### Study population

In this cross-sectional study, peripheral blood mononuclear cells (PBMC) were obtained from 39 patients with RA according to American College of Rheumatology criteria, treated at the Department of Internal Medicine II, University Hospital Würzburg, Germany, or the MVZ MED|BAYERN OST, Burghausen, Germany, either with csDMARDs only or with a TNFi with or without methotrexate, as well as from 20 healthy controls (HC, Table 1). Patients treated with biologics other than TNFi were excluded from the study. RA disease activity was assessed by determining the disease activity score of 28 joints (DAS28), as well as by measurement of the C-reactive protein (CRP) level and the erythrocyte sedimentation rate (ESR). Active disease was defined as a DAS28 ≥ 2,6. Where the DAS28 was not available, patients with an ESR ≤ 20 mm/h and a CRP ≤ 1,0 mg/dL where regarded as having an inactive disease (n = 4), patients with an elevated ESR or CRP as having an active disease (n = 2). None of the immunologically healthy donors had a personal or family history of inflammatory disease.

**Table 1:** Demographic and clinical characteristics of the study populations

Parameter	HC (*n* = 20)	csDMARD (*n* = 18)	TNFi (*n* = 21)	*p*
Chronological age, median (range) years	56.5 (21–90)	60.5 (50–84)	57 (22–73)	0.165
Female sex, n (%)	13 (65)	15 (83)	16 (76)	0.422
Age at diagnosis, median (range) years		51.0 (33–82)	42.0 (14–68)	**0.023**
Disease duration, median (range) years		8.0 (2–35)	12 (1–34)	0.241
RF positive, n (%)		12 (67)	16 (76)	0.723
ACPA positive, n (%)		17 (94)	14 (67)	**0.049**
ANA positive, n (%)		12 (67)	7 (33)	0.056
DAS28, median (range)		2.8 (1.0–6.0)	1.0 (1.0–5.3)	0.144
CRP, median (range) mg/dl	0.15 (0.15–1.45)	0.86 (0.15–5.63)	0.15 (0.15–15.20)	** < 0.001**
ESR, median (range) mm/h		13 (2–71)	7 (2–39)	**0.011**
Blood leukocytes, median (range) × 10^9^/litre	6.0 (4.3–11.3)	9.4 (3.8–14.8)	6.7 (4.2–13.8)	**0.032**
Blood lymphocytes, median (range) × 10^9^/litre	1.9 (1.2–3.4)	2.2 (1.1–5.6)	1.6 (0.4–4.0)	0.564
Active disease, n (%)		10 (56)	7 (33)	0.206
Co-medication, n (%)				
NSAID		6 (33)	3 (14)	0.255
Systemic glucocorticoids		13 (72)	6 (29)	**0.010**
Methotrexate		9 (50)	13 (62)	0.528
Leflunomide		5 (28)	0 (0)	**0.015**
Sulfasalazine		2 (11)	0 (0)	0.206
Hydroxychloroquine		2 (11)	0 (0)	0.206

*Abbreviations*: *HC* Healthy controls, *csDMARD* patients, treated with conventional synthetic disease-modifying antirheumatic drugs only, *TNFi* patients, treated with tumor necrosis factor inhibitors, *RF* rheumatoid factor, *ACPA* anti-citrullinated protein antibody, *ANA* antinuclear antibody, *DAS28* disease activity score of 28 joints, *CRP* C-reactive protein, *ESR* erythrocyte sedimentation rate, *NSAID* non-steroidal anti-inflammatory drug. Kruskal–Wallis test was performed to compare all three groups, Mann–Whitney U test to compare the csDMARD treated with the TNFi treated patients, and Fisher’s exact test was used for categorical variables

The study was approved by the ethics committee at the University of Würzburg (protocol no. 239/10).

### Quantification of T cell subsets and intracellular molecules

PBMC were isolated by using FicoLite-H (Linaris, Dossenheim, Germany) according to the manufacturer’s instructions and stimulated with phorbol-myristate-acetate (30 ng/mL) and ionomycin (1 µg/mL) in the presence of brefeldin A (5 µg/mL) (Sigma-Aldrich) for 4 h. Afterwards, they were washed with phosphate-buffered saline (PBS)/0.5% bovine serum albumine (BSA) and incubated for 20 min at room temperature in the darkness with fluorescence-labelled mouse monoclonal antibodies specific for the cell surface markers CD4, CD8, CD45RA, CD28, CD31, CD127 (IL-7 receptor alpha chain), CD25 (IL-2 receptor alpha chain), or the chemokine receptors CCR6 and CXCR3. Cells stained for cell surface markers only were washed twice with 1% BSA/PBS.

For the intracellular cytokine and transcription factor stainings, following the cell surface marker staining the cells were fixed and permeabilized using the transcription factor buffer set (BD Biosciences, San Diego, USA) according to the manufacturer’s instructions. Briefly, cells were treated with BD Pharmingen™ Fix/Perm buffer for 30 min at 4 °C in the dark. After washing with BD Pharmingen™ Perm/Wash buffer, the cells were incubated for 20 min with fluorescence-labelled mouse monoclonal antibodies specific for the transcription factor forkhead box P3 (FoxP3), the cytokines interferon-γ (IFNγ), IL-17 and IL-10, or the proliferation marker Ki67 (all antibodies from BioLegend, San Diego, CA). Finally, the cells were washed twice with BD Pharmingen™ Perm/Wash buffer.

The analyses were performed using a FACS Canto™ II flow cytometer and CellQuest™ software (BD Biosciences). Dead cells were excluded using Zombie Aqua™ Fixable Viability (BioLegend). According to phenotypic surface markers, CD45RA^+^CD28^+^ cells were characterized as naive T cells, CD45RA^−^CD28^+^ as memory T cells, CD45RA^−^CD28^−^ as effector T cells, and CD45RA^+^CD28^−^ as terminally-differentiated effector memory T cells re-expressing CD45RA (TEMRA) [35]. RTE were identified by the high expression of CD31, cells able to participate in IL-7-driven homeostatic proliferation by the expression of CD127. Treg cells were defined as FoxP3^+^. Further, naturally occurring Treg (nTreg) cells were characterized by CD25 bright expression and CD127-negativity. Representative dot plots from the flow cytometric analysis are shown in supplementary Fig. 1.

### Quantification of TREC numbers

DNA was extracted from PBMC using the QIAamp DNA Mini Kit (Qiagen, Chatsworth, CA). To remove contaminants that would interfere with polymerase chain reaction (PCR), DNA was further purified by ethanol precipitation. Signal-joint TREC concentrations were determined by quantitative real-time PCR based on the coding TREC sequence, using an iCycler reverse transcriptase-PCR system (Bio-Rad, Hercules, CA). As described in [36], we applied primers for an 82 bp DNA fragment across the remaining recombination sequence δrec/ψalpha (5’-CAC-ATC-CCT-TTC-AAC-CAT-GCT-3’ [forward] and 5’-GCC-AGC-TGC-AGG-GTT-TAG-G-3’ [reverse]). The PCR was run with 0.5 µg DNA, primers, and SYBR Green Supermix (Bio-Rad) in a final volume of 25 µl. Recombination-activating gene 2 (RAG2, primers 5’-GCA-ACA-TGG-GAA-ATG-GAA-CTG-3’ [forward] and 5’-GGT-GTC-AAA-TTC-ATC-ATC-ACC-ATC-3’ [reverse]) was used as a reference gene to normalize the quantity of DNA in peripheral lymphocytes to T cells. Each experiment was performed twice. The number of TRECs was calculated in relation to 10^4^ lymphocytes.

### Statistical analyses

Given that many variables were not normally distributed, the Kruskal–Wallis test, followed by Dunn’s multiple comparisons test, was done to compare the csDMARD-only treated with the TNFi treated RA patients and the HC.

Linear regression (least squares method) was performed to investigate the impact of the chronological age and the treatment duration on the T cell subsets representing markers of immunosenescence. The TREC counts were logarithmized for linear regression analysis.

To examine the influence of the RA diagnosis and its pharmacological treatments on the inflammatory T cell subsets, inclusive multiple linear regression was conducted using the disease status (yes/no) and the current treatment with a TNFi, methotrexate, leflunomide, non-steroidal anti-inflammatory drugs (NSAID), and systemic glucocorticoids as predictors. For the group of RA patients, we also examined the influence of the clinical parameters sex, age and disease duration, active disease (yes/no), seropositivity for rheumatoid factor (RF), anti-cyclic citrullinated peptide (anti-CCP) antibodies or anti-nuclear-antibodies (ANA), and CRP (normal/abnormal) on the T cell subsets and TRECs using multiple linear regression. Because of its non-parametric distribution we used the logarithmic transformation for the TRECs as dependent variable. CRP, RF and anti-CCP were categorized (normal/abnormal) because of high skew to the left. To control for multiple testing regarding the numerous regression models, we applied the two-stage step-up method of Benjamini, Krieger and Yekutieli [37] with a false discovery rate of Q < 0.05.

Data were analysed using SPSS version 29.0 (IBM Corp.) and GraphPad Prism version 10.4.1 (GraphPad Software, LLC). Robust regression using an M estimator was performed using rlm of the R MASS package, version 7.3–65. P values less than 0.05 were considered significant.

## Results

### Patient characteristics

To investigate the progression of immunosenescence in differently treated RA patients we compared 21 patients treated with TNFi (15 Etanercept, 6 Adalimumab, 13 in combination with methotrexate, 8 TNFi only) with 18 patients, treated with csDMARDs only, and 20 healthy controls (HC, Table 1). Whereas the chronological age at the time of blood withdrawal did not differ between the study groups, the TNFi treated patients had a younger age at diagnosis and a longer overall disease duration. Regarding disease activity, there was no difference in the DAS28 or the proportion of patients with an active disease between the TNFi- and the csDMARD-treated patients. Concerning laboratory parameters, however, the TNFi-treated patients had lower values for CRP, ESR and blood leukocyte counts, the latter possibly caused by a higher proportion of patients receiving systemic glucocorticoids within the csDMARD treated group. The further co-medication consisted of methotrexate and NSAIDs within both treatment groups, whereas leflunomide, sulfasalazine and hydroxychloroquine was restricted to the csDMARD-only treated patients.

###  T helper cells of csDMARD-only treated RA patients display features of an advanced immunosenescence, compared to TNFi treated patients or HC

**Fig. 1:**
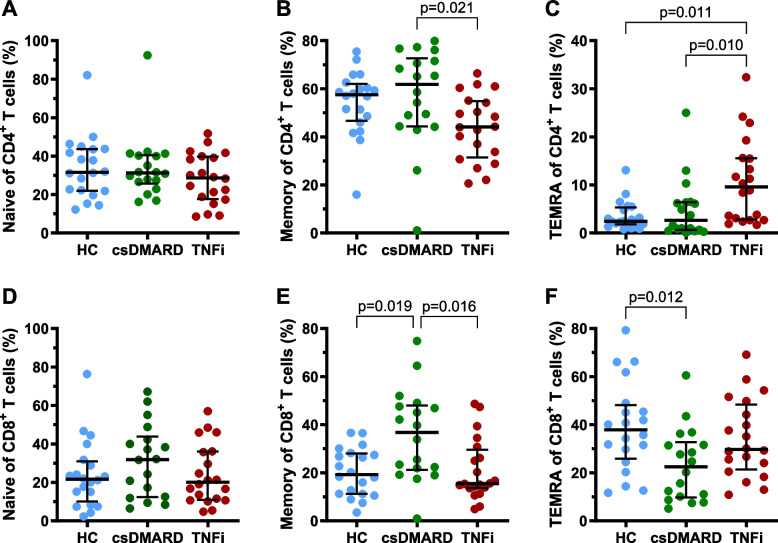
Naive, memory and terminally-differentiated effector memory T cells (TEMRA) in differently treated rheumatoid arthritis patients. Proportions of naive, memory and terminally-differentiated effector memory T cells re-expressing CD45RA (TEMRA) in rheumatoid arthritis (RA) patients, treated with conventional synthetic disease modifying antirheumatic drugs only (csDMARD) or tumor necrosis factor inhibitors (TNFi), compared to healthy controls (HC). The proportions of naive CD4^+^ and CD8^+^ T cells did not differ between the csDMARD and the TNFi treated patients (**A**, **D**). TNFi treated patients, however, had lower proportions of CD4^+^ and CD8^+^ memory T cells than the csDMARD-only treated patients (**B**, **E**). On the other hand, the CD4^+^ TEMRA cells were elevated in TNFi treated patients (**C**). Bars represent median percentages and interquartile range. Kruskal–Wallis test was performed, followed by Dunn’s test to compare between the study populations

As ageing of the immune system is characterized by a decrease in naive T cells and an increase in memory T cells and TEMRA cells [1], we determined the relative amounts of these T cell subtypes in RA patients, treated with csDMARDs only or with TNFi, and compared them to HC (Fig. 1). Whereas the proportions of naive CD4^+^ and CD8^+^ T cells did not differ between the study groups (Fig. 1A, D), TNFi treated patients displayed lower proportions of CD4^+^ and CD8^+^ memory T cells than the csDMARD-only treated patients (Fig. 1B, E). The TNFi treated patients, however, displayed increased proportions of CD4^+^ TEMRA cells, whereas the CD8^+^ TEMRA cells were slightly reduced within the csDMARD-only treated patients, compared to the HC (Fig. 1C, F).

**Fig. 2:**
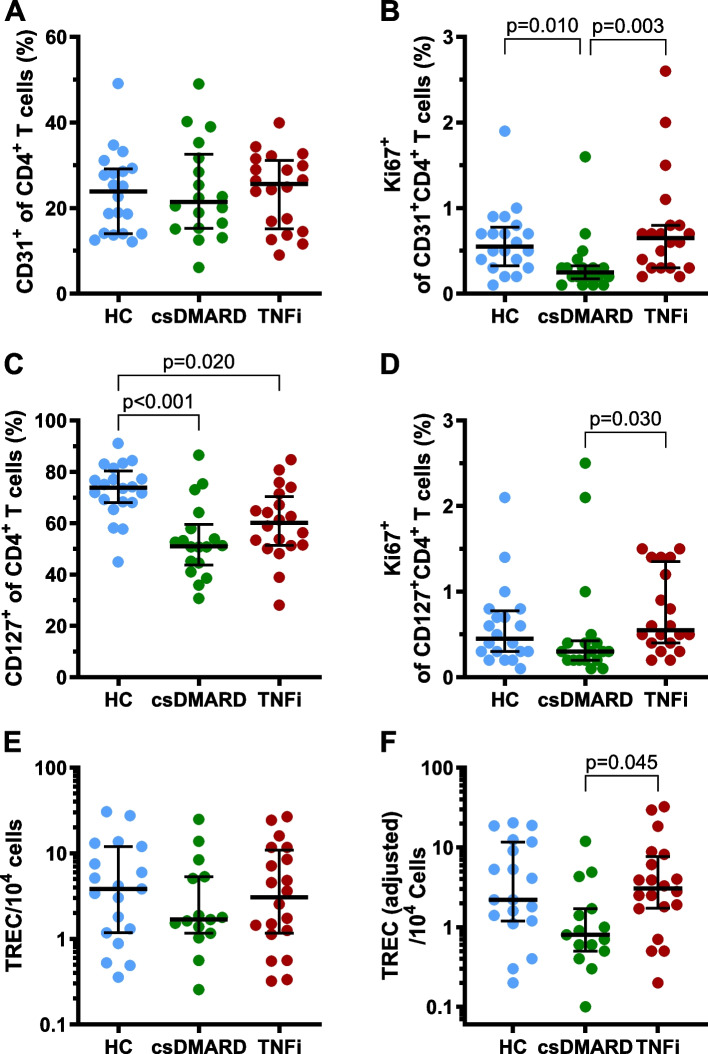
Thymic function and peripheral homeostatic T cell proliferation in differently treated RA patients. Proportions of CD31^+^ recent thymic emigrants (RTE), CD127^+^ T cells able to participate in homeostatic T cell proliferation, proliferating Ki67^+^ cells of the CD31^+^CD4^+^ and CD127^+^CD4^+^ T cells, and T cell receptor excision circle (TREC) containing cells in RA patients, treated with csDMARDs only or an additional or exclusive TNFi, compared to HC. Whereas the RTE did not differ significantly between the study populations (**A**), the rate of proliferating RTE was significantly lower in the csDMARD treated RA patients than in the TNFi treated patients or HC (**B**). The CD127^+^ proportions of the CD4^+^ T helper cells were reduced in both RA groups, compared to the HC (**C**), whereas the rate of proliferating CD127^+^CD4^+^ T cells was higher within the TNFi treated than the csDMARD-only treated RA patients (**D**). The proportion of TREC-containing cells was slightly reduced within the csDMARD-only treated patients (**E**). When the TREC counts were adjusted for the differing peripheral proliferation rates of the T cells, the reduced thymic function of the csDMARD-treated patients became even more obvious (**F**). Bars represent median percentages and interquartile range. Kruskal–Wallis test was performed, followed by Dunn’s test to compare between the study populations

Since, in contrast to children and adolescents [36], the age-dependent alterations in naive and memory T cells in adults are of minor extent, immunosenescence in our patient cohorts may be better reflected by other parameters. Therefore, we determined the frequency of RTE, characterized by the expression of CD31 [38], and the proportion of CD127^+^ cells, being able to participate in peripheral IL-7-driven homeostatic T cell proliferation. Comparing csDMARD-only and TNFi-treated RA patients, a statistically non-significant tendency towards higher proportions of CD31^+^CD4^+^ T cells, CD127^+^CD4^+^ T cells and TREC containing cells within the TNFi treated group was found (Fig. 2A, C, E). Both treatment groups exhibited reduced proportions of CD127^+^CD4^+^ T cells, compared to the HC (Fig. 2C). Comparing the rate of proliferating RTE or CD127^+^ T cells, significantly reduced numbers of Ki67-expressing cells were found within the csDMARD-only treated patients, whereas the TNFi treated patients displayed Ki67^+^ proportions comparable to the HC (Fig. 2B, D). As peripheral proliferation of naive T cells leads to a dilution of the non-replicating TRECs [39], TREC numbers were adjusted for the proliferation rate of the CD31^+^CD4^+^ T cells, resulting in significantly lower TREC numbers in the csDMARD-only treated patients compared to TNFi treated patients (Fig. 2F).

**Fig. 3:**
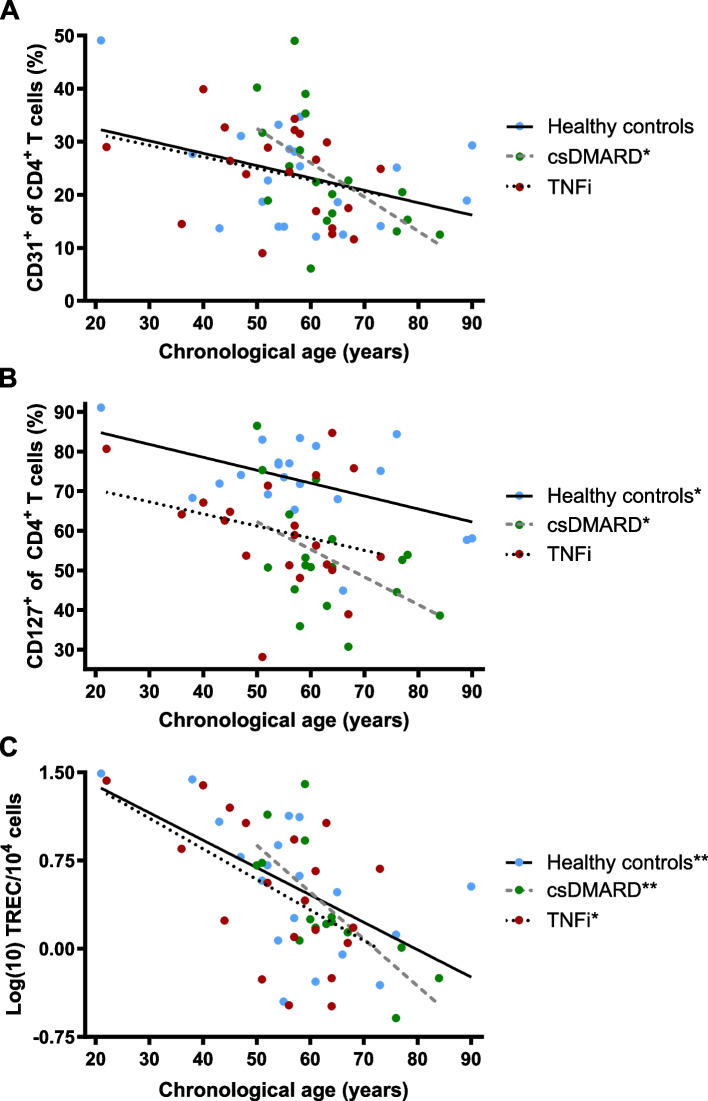
Velocity of non-inflammatory immunoscenescence in differently treated RA patients. Linear regression analyses of the proportions of CD31^+^ RTE, the CD127^+^ T cells able to participate in homeostatic T cell proliferation, and the logarithmized T cell receptor excision circle containing cells (TREC) against the patients’ ages, separated by csDMARD-only or TNFi treated RA patients and HC. Whereas the age-dependent decrease of the RTE (**A**), the CD127^+^ T cells (**B**) and the TREC counts (**C**) is similar between TNFi treated RA patients and HC, the csDMARD treated patients show an accelerated decline of the respective parameters. Asterisks indicate a statistically significant (* *p* < 0.05, ** *p* < 0.01) non-zero slope of the respective regression line

To compare the rate of the immunological ageing between the patient groups and the HC group, linear regression analyses were performed to plot the cross-sectional peripheral T cell data against the chronological age of the patients at blood withdrawal (Fig. 3, Table 2). Whereas the age-dependent decline of the proportions of CD31^+^ RTE (Fig. 3A) and CD127^+^CD4^+^ T cells (Fig. 3B) was only modest within the TNFi-treated patients and HC, the csDMARD-only treated patients displayed a significant age-dependent decrease of the respective T helper cell populations. The differences in the slopes of the regression lines, however, did not reach statistical significance. Nevertheless, the age-dependent changes of almost all non-inflammatory parameters of immunosenescence within the TNFi-treated RA patients were similar to the HC, whereas the csDMARD-only treated patients displayed signs of an accelerated immunosenescence (Table 2).

**Table 2:** Velocity of non-inflammatory immunoscenescence in differently treated RA patients

	**HC**	**csDMARD**	**Velocity of immuno-scenescence**	**TNFi**	**HC**
Naive CD4^+^ T cells		− 2.3	>	− 1.3	− 0.2
Memory CD4^+^ T cells		5.2	>	− 2.2	0.0
CD31^+^ of CD4^+^ T cells		− 6.5*	>	− 2.2	− 2.3
CD127^+^ of CD4^+^ T cells		− 7.0*	>	− 3.1	− 3.3*
CD127^+^CD31^+^ of CD4^+^ T cells		− 4.9*	>	− 3.3**	− 3.2*
Ki67^+^ of CD31^+^CD4^+^ T cells	0.02	0.03	=	− 0.03	
Ki67^+^ of CD127^+^CD4^+^ T cells		− 0.21	>	0.13	0.00
TREC^a^		− 0.40**	>	− 0.26*	− 0.23**

Velocity of non-inflammatory immunoscenescence in RA patients, treated with conventional synthetic disease modifying antirheumatic drugs (csDMARDs) only or tumor necrosis factor inhibitors (TNFi), compared to healthy controls (HC). Depicted is the slope of the linear regression of the respective parameters against the chronological age of the patients at blood withdrawal [‰ change per year]. Values for the HC are depicted in the left HC column when they are similar to the csDMARD treated RA patients and in the right HC column, when similar to the TNFi treated RA patients. Asterisks indicate a statistically significant (* *p* < 0.05, ** *p* < 0.01) non-zero slope of the respective linear regression

^a^The TREC counts were logarithmized for linear regression analysis [Log10(TREC/10^4^ cells/10 years)]

Taken together, markers of thymic function and peripheral T cell homeostasis showed comparable age-related alterations between TNFi-treated RA patients and HC, whereas the csDMARD-only treated patients displayed signs of an accelerated ageing of the immune system.

### csDMARD-only treated RA patients display reduced proportions of unspecifically activated inflammatory effector and memory T cells

Apart from the shift of naive to antigen-experienced T cells of memory and effector type, the decline in RTE, and the changes in homeostatic T cell proliferation, immunosenescence has been shown to be associated with inflammageing, characterized by the accumulation of terminally differentiated effector memory T cells, either specific for self-antigen or for latent viruses, having a low activation threshold, and generating an inflammatory environment, whereas the functional capacity of regulatory T cells subsides [14, 15].

As not all of our RA patients had an inactive disease, associations between RA disease activity and proportions of inflammatory and regulatory T cell subsets were investigated (Suppl. Table 1). The proportions of inflammatory and regulatory T cell subsets did not differ between patients with an inactive disease and those with clinical or laboratory signs of disease activity. Thus, as our study subjects did not show any clinical sign of infection at the time the blood was drawn, the measured proportions of inflammatory cytokine secreting effector, memory or TEMRA cells could be regarded as unspecific inflammation within the context of immunosenescence.

**Fig. 4:**
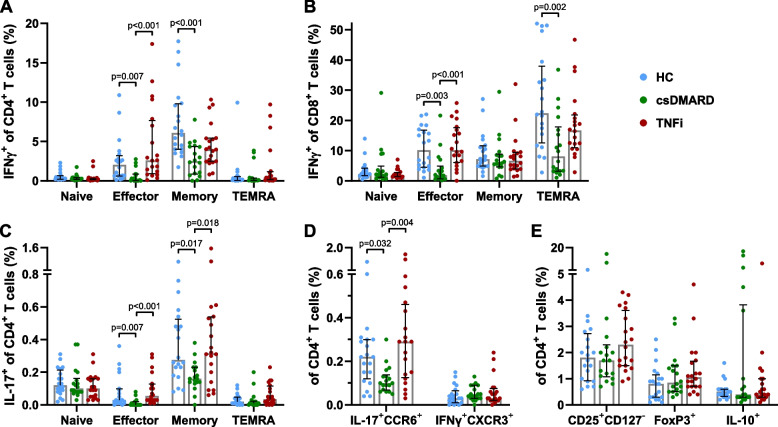
Proportions of inflammatory cytokine secreting and regulatory T cells in differently treated RA patients. Inflammatory cytokine secreting and regulatory T cells in RA patients, treated with csDMARDs only or TNFi, compared to HC. csDMARD-only treated patients showed significantly reduced proportions of IFNγ^+^CD4^+^ effector and memory T cells (**A**), IFNγ^+^CD8^+^ effector and TEMRA T cells (**B**), IL-17^+^CD4^+^ effector and memory T cells (**C**), as well as IL-17^+^CCR6^+^ Th17 cells (**D**). The proportions of CD25^+^CD127^−^, FoxP3^+^ or IL10^+^ T helper cells did not differ between the study groups (**E**). Bars represent median percentages and interquartile range. Kruskal–Wallis test was performed, followed by Dunn’s test to compare between the study populations

Compared to HC, csDMARD-only treated RA patients displayed significantly reduced proportions of IFNγ^+^CD4^+^ effector and memory T cells (Fig. 4A), IFNγ^+^CD8^+^ effector and TEMRA T cells (Fig. 4B), IL-17^+^CD4^+^ effector and memory T cells (Fig. 4C), and IL-17^+^CCR6^+^ Th17 cells (Fig. 4D). The proportions of inflammatory cytokine secreting T cells in the TNFi-treated patients, however, did not significantly differ from HC (Fig. 4A-D).

As 62% of the TNFi treated patients received a co-medication with methotrexate (Table 1), the proportions of inflammatory cytokine secreting effector T cells were compared between patients treated with csDMARDs only, TNFi only, or treated with TNFi in combination with methotrexate (Suppl. Fig. 2). The TNFi-only treated patients displayed increased proportions of IFNγ^+^CD4^+^, IFNγ^+^CD8^+^, IL-17^+^CD4^+^ effector T cells and Th17 cells, compared to the HC or to patients treated with an additional or exclusive csDMARD. Thus, patients treated with TNFi in combination with methotrexate most closely resembled the HC.

Taken together, csDMARD-only treated RA patients display reduced proportions of unspecifically activated, IFNγ- or IL-17-secreting effector and memory T cells.

### Unspecifically activated, IFNγ-secreting CD8^+^ TEMRA cells represent inflammageing and are induced by TNFi treatment

**Fig. 5:**
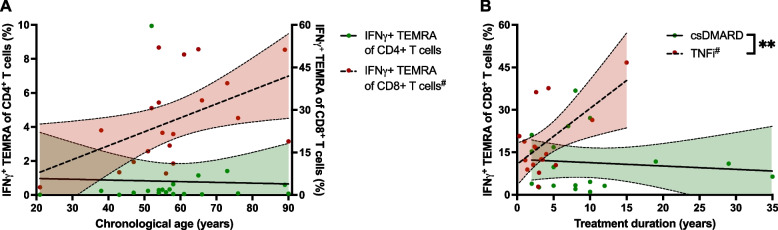
Proportions of IFNγ-secreting TEMRA cells depending on age or treatment duration with csDMARDs or TNFi. Linear regression analyses of the proportions of IFNγ-secreting CD4^+^ or CD8^+^ terminally-differentiated effector memory T cells re-expressing CD45RA (TEMRA) against the HCs chronological age or the treatment duration, separated by csDMARD-only and TNFi treated RA patients. Whereas the IFNγ^+^CD8^+^TEMRA cells increase with age, probably representing inflammageing, the IFNγ^+^CD4^+^TEMRA cells do not (**A**). Regarding treatment duration, the proportions of IFNγ^+^CD8^+^TEMRA cells increase over time in TNFi treated patients, whereas they do not change in csDMARD-only treated patients (**B**). Depicted are the respective regression lines with 95% confidence bands. ^# ^indicates a statistically significant (*p* < 0.05) non-zero slope of the respective regression line. Asterisks indicate a statistically significant (** *p* < 0.01) difference in the slope of the respective regression lines

As inflammageing is characterized by terminally differentiated effector T cells, generating an inflammatory environment, we investigated the associations between the IFNγ- or IL-17-secreting TEMRA cells and the chronological age within the HC population. Linear regression analyses revealed, that the IFNγ^+^CD8^+^TEMRA cells increase with age (Fig. 5A), whereas the IFNγ^+^ or IL-17^+^CD4^+^TEMRA cells do not. This suggests that the numbers of IFNγ^+^CD8^+^TEMRA cells indeed represent inflammageing.

To figure out if the differing proportions of inflammatory CD8 TEMRA cells between the csDMARD-only and the TNFi treated patients are due to the medication, or whether they are features of the underlying rheumatic disease requiring the particular treatment forms, linear regression analyses of the proportions of IFNγ^+^CD8^+^TEMRA cells against the treatment duration were performed (Fig. 5B). Surprisingly, whereas there was no association between the proportions of IFNγ^+^CD8^+^TEMRA cells and the treatment duration in patients treated with csDMARDs only, the cell-population was higher in patients on a long-term TNFi-treatment than in patients with a recent TNFi initiation. As there was considerable variability in the proportions of IFNγ^+^CD8^+^TEMRA cells and we only had a limited number of patients on a stable long-term treatment, we also performed robust regression analyses, confirming the age-dependent increase in the IFNγ^+^CD8^+^TEMRA cells of the HC population (β = 0.52, *p* = 0.026), as well as the difference in the dependence on the treatment duration between the csDMARD-only and the TNFi treated patients (β = 2.10, *p* = 0.011). The higher proportions of IFNγ-secreting CD8 TEMRA cells in the long-term TNFi treated patients were due to higher amounts of TEMRA cells, as well as higher IFNγ-positive proportions of these cells (data not shown).

Regarding the regulatory T cell subsets, we neither found any difference between the RA patients treated with differing DMARD regimes (Fig. 4E), nor any significant alterations with the patients’ ages or the treatment duration.

Taken together, the IFNγ^+^CD8^+^TEMRA cells increase with age, possibly representing inflammageing. In addition, the IFNγ^+^CD8^+^TEMRA cells appear to increase with the duration of a TFNi treatment.

### TNFi treatment is associated with increased proportions of inflammatory T cell subsets in RA patients

To further investigate the effects of the RA diagnosis and the differing treatment modalities on the proportions of cytokine-producing inflammatory T cells, multiple linear regression analyses were performed including the disease status (yes/no) and the current treatment with TNFi, methotrexate, leflunomide, NSAIDs, and systemic glucocorticoids as independent variables (Suppl. Table 2). Interestingly, multiple regression analysis resulted in a negative association between an RA diagnosis and the proportion of IFNγ^+^CD4^+^ memory T cells and positive associations between a TNFi treatment and the proportions of IFNγ^+^CD8^+^ effector T cells and Th17 cells, whereas a treatment with methotrexate or leflunomide did not contribute significantly to the regression models.

In order to identify additional parameters influencing the markers of T cell immunosenescence in RA patients, multiple regression analyses were performed including current treatment with TNFi, sex, age, disease duration, disease activity, RF status, anti-CCP-antibody status, ANA status and CRP as independent variables, and the T cell subpopulations or TREC count as dependent variables (Suppl. Table 3). Regarding clinical parameters, a positive association between the inflammatory activity of the RA and the proportion of Th1 cells (IFNγ^+^CXCR3^+^CD4^+^ T cells, regression coefficient 0.07, *p* = 0.003) and a negative association between age and TREC counts (regression coefficient -0.066, *p* = 0.003) was found.

Altogether, the effect of a TNFi-treatment on the inflammatory T cell-subpopulations was larger than the effects of the co-medication or the clinical parameters.

## Discussion

RA is a chronic inflammatory disease that has been associated with premature immunosenescence. In general, T cell immunosenescence could be divided into two categories: on the one hand, the non-inflammatory age-associated changes, essentially based on a reduced thymic output. This results in a diminished proportion of CD31^+^ RTE, lower TREC counts within naive T cells, a reduced ratio of naive to memory T cells, and an enhanced peripheral homeostatic proliferation of naive T cells to counteract the diminished thymic output. On the other hand, immunosenescence is characterized by an increase in unspecifically activated, terminally differentiated inflammatory T cells, as well as a numeric and functional decline of regulatory T cells, resulting in a bias towards less specific and more inflammatory immunity (inflammageing). The effects of the currently available treatment modalities on the premature immunosenescence in RA have not been investigated until now. This study was performed to compare the effects of a pharmacological treatment with csDMARDs only or an additional or exclusive TNFi on the parameters of T cell immunosenescence in RA.

Regarding the non-inflammatory parameters of immunosenescence, we found some indications that TNFi treatment decreases phenotypical features of the ageing of the immune system in RA patients more effectively than treatment with csDMARDs alone. Along that line, the TNFi treated patients presented with lower numbers of memory T cells, whereas the proportions of naive T cells did not differ between the patient cohorts. In this context, it must be mentioned that TNF can not only promote the activation and proliferation of naive and effector T cells, but also induces apoptosis of activated effector T cells [40]. Accordingly, the relative proportions of CD4 and CD8 effector T cells were elevated within the TNFi-treated RA patients of our study, a finding which might partially explain the differences in the relative proportions of memory and TEMRA cells between the csDMARD-only and the TNFi-treated patients.

CD127 was studied to estimate the susceptibility of the T cells to IL-7 as a factor of homeostatic proliferation [41, 42]. Several studies have shown that despite an age-dependent decline in thymic function, which would require a prolonged survival or increased peripheral homeostatic proliferation of naive T cells, the expression of IL-7 as well as the expression of the specific α-chain of its receptor (CD127) decline with age [43–45]. In our study, the RA patients presented with reduced proportions of CD127^+^ CD4 T cells compared to HC, with a slight tendency towards normalization in the TNFi treated patients. Consistent with the literature, the proportions of CD127^+^ CD4 T cells decreased with age, which was pronounced in csDMARD-only treated patients. Similarly, lower numbers of CD4^+^ RTE with higher age was stressed within the csDMARD group. Regarding proliferation of the RTE or CD127^+^ T cells, the proportions of Ki67 expressing cells were significantly diminished within the csDMARD-only treated patients, whereas the TNFi treated patients displayed proportions of Ki67^+^ cells comparable to the HC. Furthermore, the TREC counts in TNFi treated patients were comparable to HC, whereas the csDMARD-only treated patients displayed diminished TREC counts. As peripheral proliferation leads to dilution of TRECs, the slightly reduced TREC count of the csDMARD-only treated patients despite a reduced peripheral proliferation indicates that this cohort features the lowest thymic output.

It needs to be noted, however, that regarding the non-inflammatory parameters of immunosenescence, age-related changes were less significant in our cohorts than expected. Furthermore, the adjustment of the TREC counts to the differing peripheral proliferation rates of the RTE (Fig. 3F) needs to be interpreted with caution, as Ki67-expression only refers to one timepoint of analysis, whereas TRECs rather show the replicative history over a cells life. Therefore, despite some indications that TNFi treatment is more effective in normalizing the accelerated non-inflammatory immunosenescence in RA than a treatment with csDMARDs only, definite conclusions cannot be drawn from the data currently available.

One reason for the only minor age-related alterations within our cohorts could be, that the immunosenescence associated changes become less pronounced with increasing age. In a study by Fessler et al., significantly reduced TREC numbers in RA patients up to an age of 45 years were found [46], whereas the TREC numbers in older RA patients did not differ from age-matched HC. In our cohort only 12% of the patients where at an age less than 45 years which may – besides the high interindividual variability – explain the relatively low age-dependency of the measured parameters.

An additional parameter, commonly used as an indicator of increased replicative history and cellular senescence, is telomere length. We, however, were not able to detect any age dependence or differences in telomere length, neither between the RA patients and the HC, nor between the differently treated patient cohorts (data not shown). The reason might be a methodological problem, as the qPCR we performed to determine telomere length also failed to detect shortened telomeres in RA patients in other studies [17, 21], whereas shortened telomeres in RA patients were found when the terminal restriction fragment methodology was applied [22].

In contrast to the only minor differences between the treatment groups regarding the non-inflammatory parameters of immunosenescence, substantial differences in the parameters of inflammageing were found. First of all, the TNFi-treated patients displayed elevated proportions of CD4 TEMRA cells, compared to the csDMARD-only treated RA patients or HC. Comparing the proportions of unspecifically activated, inflammatory cytokine secreting T cells between RA patients treated with either csDMARDs only or an additional or exclusive TNFi, it seemed that a csDMARD treatment is associated with reduced proportions of IFNγ- or IL-17-secreting effector, memory and TEMRA T cells. Performing multiple linear regression analyses to dissect the influence of the RA and its treatment options on the inflammatory cytokine secreting T cells, the regression models revealed a negative association between the RA disease occurrence (or the DMARD-treatment in its entirety as no untreated patients were included) and a positive association of TNFi treatment with the proportions of inflammatory T cells, such as IFNγ^+^CD8^+^ T cells and Th17 cells. This increase in unspecifically activated cytokine producing T cells upon blocking TNF possibly represents some kind of ‘compensatory immunological switch’, which might contribute to the induction or exacerbation of secondary autoimmune phenomena, such as systemic lupus erythematosus or psoriasis, in TNFi treated patients [47].

Regarding the analysis of inflammatory T cells, the disease activity of the RA needs to be taken into account. In contrast to some other studies on immunosenescence in rheumatic diseases, we also included patients with an active disease. However, in depth analysis showed, that neither clinical disease activity, nor the disease associated laboratory parameters were associated with changes of the investigated parameters. This is possibly due to the fact, that all patients were on a long-term DMARD therapy, and thus 60% of the study subjects with an active RA (DAS28 ≥ 2.6) had a low disease activity (DAS28 < 3.2). Furthermore, the csDMARD-only treated patients, exhibiting significantly reduced proportions of inflammatory cytokine producing T cells, had significantly higher CRP- and ESR-values than the TNFi-treated patients and a tendency towards a higher DAS28. Taken together, it is very unlikely that the observed differences in the inflammatory T cell subpopulations between the csDMARD and the TNFi treated patients were driven by differences in disease activity. These findings are in accordance with a recently published study on the effects of tofacitinib treatment on immunosenescence in RA, which also did not find any relevant impact of the RA disease activity on T cell senescence [48]. The study also included patients treated with TNFi and other DMARDs (mainly methotrexate) and did not find any significant differences between these two treatment groups, untreated patients and HC. However, the approach to define immunosenescence was quite different from our investigation, and we did not include patients treated with targeted synthetic DMARDs like tofacitinib. The finding that tofacitinib activates immunosenescence pathways while simultaneously inhibiting T cell effector functions, however, resembles our findings in the csDMARD-treated patients.

Concerning the patients’ concomitant medication, a major difference between our two treatment groups was the increased usage of glucocorticoids in the csDMARD-only group, which might have been expected to contribute to the reduced amounts of inflammatory cytokine secreting T cells. However, neither the multiple linear regression analyses, nor direct comparison of the patients receiving systemic glucocorticoids or not (data not shown) indicated any relevant impact of this co-medication. The multiple regression analyses performed rather show that the influence of a TNFi treatment on the inflammatory T cell subpopulations is larger than the influence of the co-medications or clinical parameters of disease. Nevertheless, a limitation of the study is the differences in some baseline characteristics between the RA treatment groups, possibly influencing the measured parameters. Furthermore, we do not have data on the prevalence of chronic viral infections, e.g. with cytomegalovirus or Ebstein-Barr virus, in our cohorts. As these infections are known to drive immunosenescence, we cannot rule out a relevant impact on the measured parameters.

As we performed a cross-sectional analysis, one could argue that the differences in the inflammatory T cell subpopulations between the csDMARD and the TNFi treated patients do not reflect effects of the differing treatment regimes, but diverse RA disease courses, requiring the respective treatment forms. The apparent correlation between the TNFi treatment duration and the proportions of IFNγ producing CD8 TEMRA cells, however, rather suggests an association with the medication than with the underlying disease. The age-associated increase of the IFNγ^+^CD8^+^TEMRA cells within the HC population (Fig. 5A) supports the assumption that this cell population actually represents inflammageing. Furthermore, it has been shown that the proportion of IFNγ-expressing T cells, following incubation with PMA and ionomycin, is higher in CD8 T cells expressing the T cell senescence marker KLRG1 than in KLRG1-negative T cells [49]. Our finding, that preferentially CD8^+^ TEMRA cells seem to represent inflammageing, whereas CD4^+^ TEMRA cells do not, is consistent with the literature [8, 14, 49].

In summary, the T cell specific immunosenescence features of patients treated with a TNFi in combination with csDMARDs most closely resemble that of HC, which may indicate a reconstitution of the T cell immune system to physiological levels. However, the question arises, whether the reduced numbers of inflammatory cytokine secreting T cells in the csDMARD treated patients constitute some kind of protection from inflammageing associated cardiovascular disease [50], cognitive impairment [33, 51] and additional autoimmune manifestations in RA, or whether the increased numbers of IFNγ^+^CD8^+^ cytotoxic T cells and Th17 cells in the TNFi treated patients renders them less prone to experience severe viral infections than RA patients with other treatment regimens [52].

## Conclusions

Whereas a TNFi treatment seems to counteract the non-inflammatory aspects of immunosenescence regarding T cell homeostasis, it induces increasing proportions of terminally-differentiated, cytokine-producing effector memory T cells, requiring awareness as possibly driving solid organ ageing and contributing to secondary autoimmune phenomena in RA, such as the development of a systemic lupus erythematosus or psoriasis.

## Supplementary Information

Supplementary Material 1: **Supplementary Figure 1**. Gating strategy of the flow cytometric analyses performed. According to phenotypic surface markers, CD45RA^+^CD28^+^ cells were characterized as naive T cells, CD45RA^-^CD28^+^ cells as memory T cells, CD45RA^-^CD28^-^ cells as effector T cells, and CD45RA^+^CD28^-^ cells as terminally-differentiated effector memory T cells re-expressing CD45RA (TEMRA). The proportions of IFNγ-/IL-17-expressing naive/effector/memory or TEMRA cells were determined (**A**). CD4^+^ RTE were identified by the expression of CD31, cells able to participate in IL-7-driven homeostatic proliferation by the expression of CD127. Proliferation of RTE or CD127^+^CD4^+^ T cells was determined by Ki67 (**B**). Th17 cells were determined by the expression of IL-17 and CCR6, Th1 cells by the expression of IFN and CXCR3 (**C**). Treg cells were defined as FoxP3^+^. Naturally occurring Treg (nTreg) cells were characterized by CD25 bright expression and CD127-negativity. Furthermore, the proportion of CD4^+^ T cells expressing IL-10 was determined (**D**). **Supplementary Figure 2**. Proportions of inflammatory cytokine secreting effector T cells in differently treated RA patients. Inflammatory cytokine secreting effector T cells in RA patients, treated with csDMARDs-only, TNFi-only or a combination of a TNFi and a csDMARD (methotrexate), compared to HC. csDMARD-only treated patients showed significantly reduced numbers of IFNγg CD4 , IFNγ CD8 (**A**) or IL-17 CD4 (**B**) effector T cells, compared to HC. TNFi-only treated patients instead displayed increased numbers of the respective effector T cells. Patients treated with the combination of a TNFi and methotrexate most closely resemble the HC. Bars represent median percentages and interquartile range. Kruskal-Wallis test was performed, followed by Dunn’s test to compare between the study populations.

Supplementary Material 2: **Supplementary Table 1**. Inflammatory and regulatory T cell subsets in inactive and active patients. **Supplementary Table 2**. Influence of the RA and its pharmacological treatments on the inflammatory T cell subpopulations / the TREC count. **Supplementary Table 3**. Associations between clinical parameters of the RA and the inflammatory T cell subpopulations / the TREC count.

## Data Availability

The datasets analysed during the current study are available from the corresponding author on reasonable request.

## Abbreviations

ANA: Anti-nuclear-antibody
BSA: Bovine serum albumine
CCP: Cyclic citrullinated peptide
CD: Cluster of differentiation
CRP: C-reactive protein
csDMARD: Conventional synthetic disease-modifying antirheumatic drug
DAS28: Disease activity score of 28 joints
DNA: Deoxyribonucleic acid
ESR: Erythrocyte sedimentation rate
FoxP3: Forkhead box P3
HC: Healthy control
IFNγ: Interferon-γ
IL: Interleukin
MHC: Major histocompatibility
NSAID: Non-steroidal anti-inflammatory drug
nTreg: Naturally occurring regulatory T cells
PBMC: Peripheral blood mononuclear cells
PBS: Phosphate-buffered saline
PCR: Polymerase chain reaction
RA: Rheumatoid arthritis
RAG2: Recombination-activating gene 2
RF: Rheumatoid factor
RTE: Recent thymic emigrant
TCR: T cell receptor
TEMRA: Effector memory T cells re-expressing CD45RA
Th: T helper cell
TNFi: Tumor necrosis factor inhibitor
TREC: T cell receptor excision circle
Treg cells: Regulatory T cells
