# Supplementary figures and images for: Diverging effects of tumor necrosis factor inhibitors and conventional synthetic disease-modifying antirheumatic drugs on immunosenescence and inflammageing in rheumatoid arthritis: a cross-sectional analysis
Source: Immun Ageing. 2025 May 22;22:21. doi: 10.1186/s12979-025-00508-w (PMC12096643; doi:10.1186/s12979-025-00508-w)

A

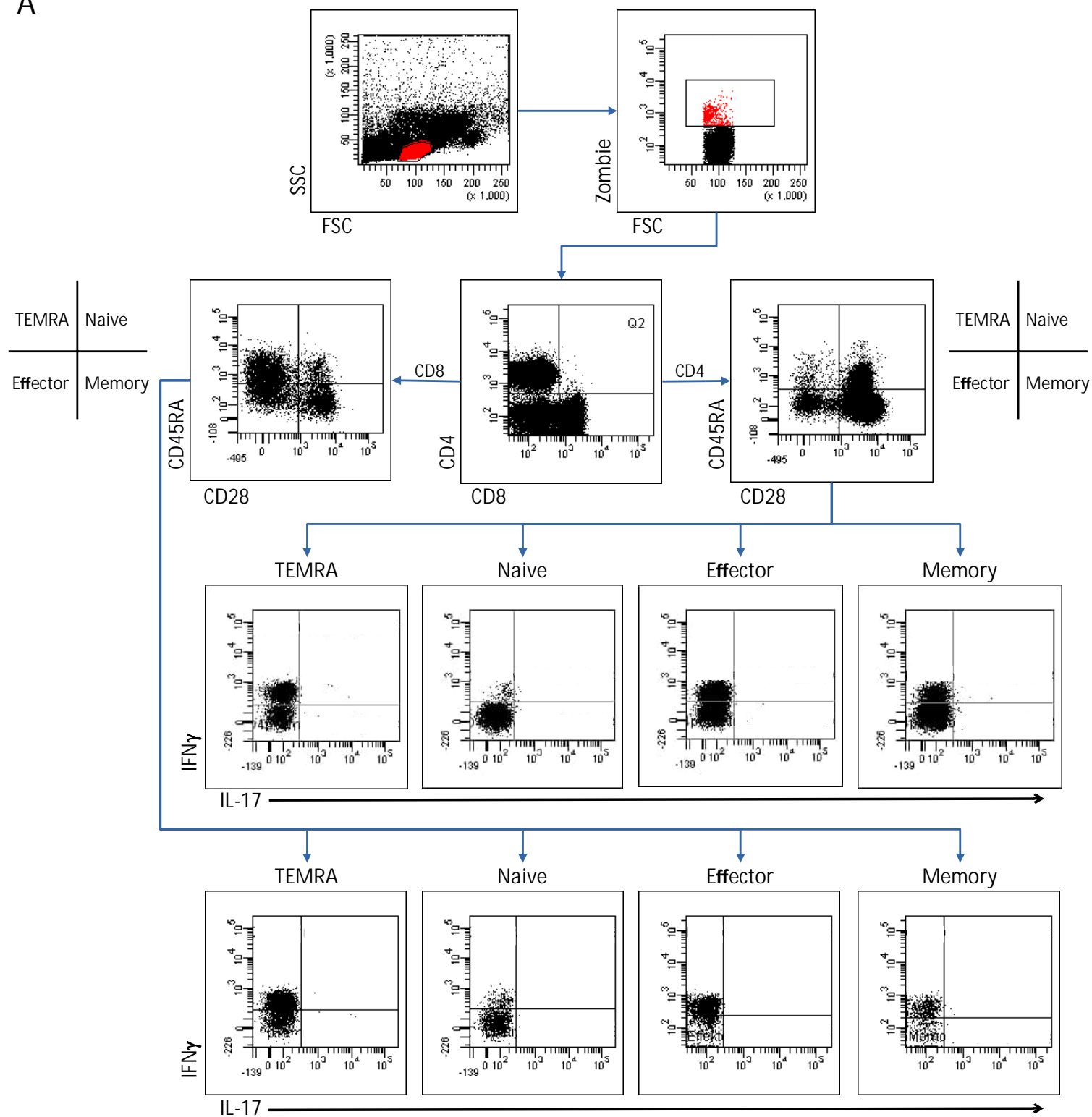

B

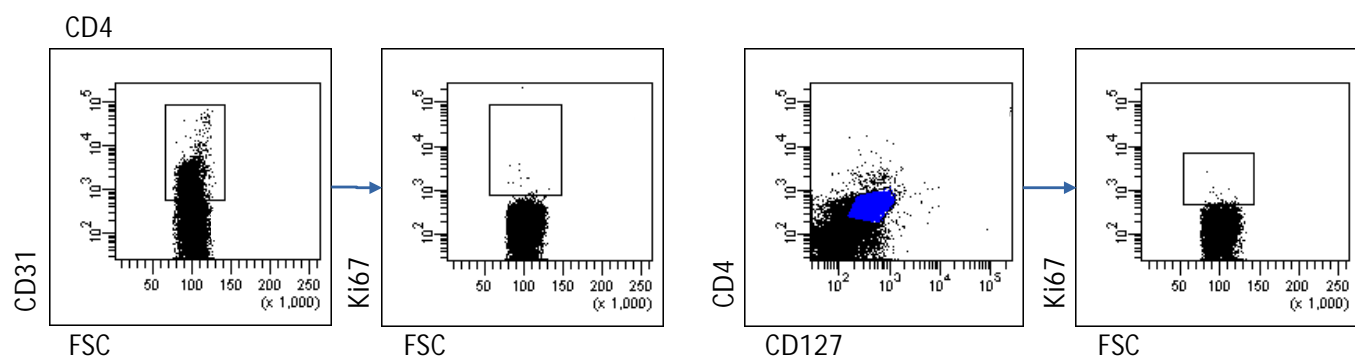

C

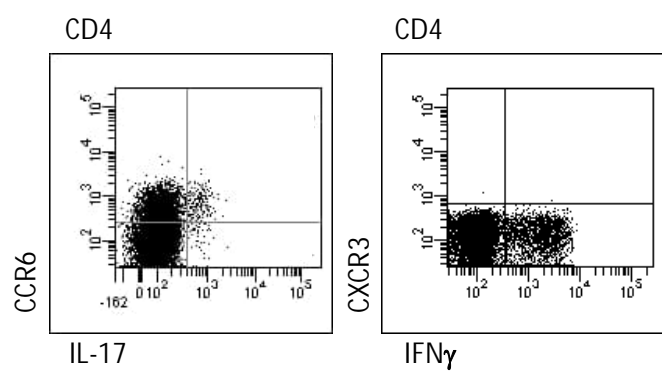

D

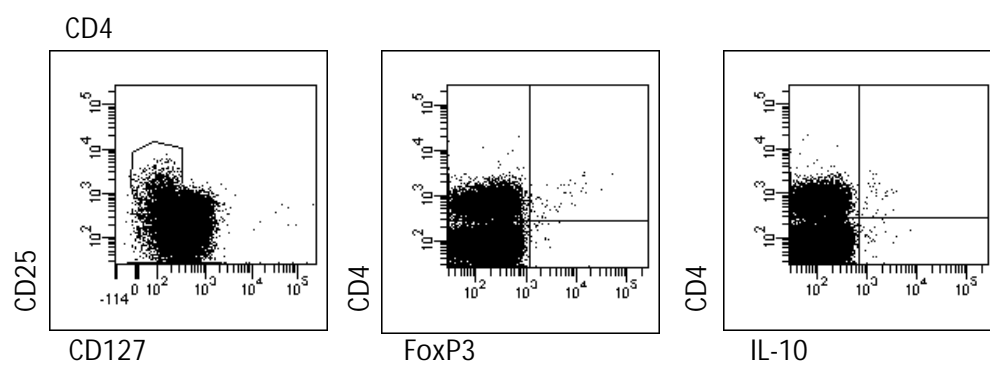

**A**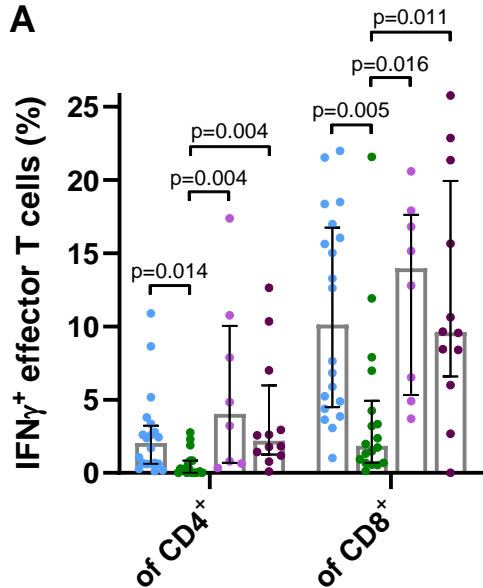**B**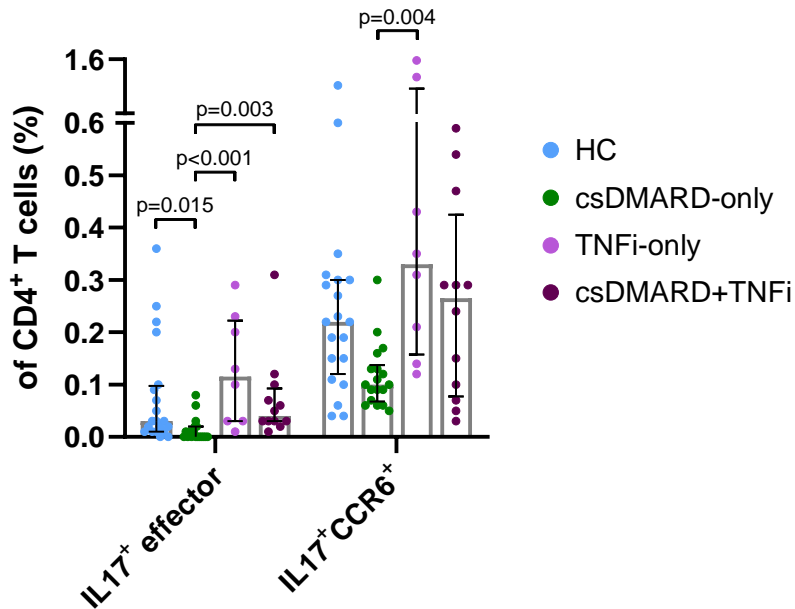

Supplement: Supplementary file 1 — Supplementary Material 1: Supplementary Figure 1. Gating strategy of the flow cytometric analyses performed. According to phenotypic surface markers, CD45RA+CD28+ cells were characterized as naive T cells, CD45RA-CD28+ cells as memory T cells, CD45RA-CD28- cells as effector T cells, and CD45RA+CD28- cells as terminally-differentiated effector memory T cells re-expressing CD45RA (TEMRA). The proportions of IFNγ-/IL-17-expressing naive/effector/memory or TEMRA cells were determined (A). CD4+ RTE were identified by the expression of CD31, cells able to participate in IL-7-driven homeostatic proliferation by the expression of CD127. Proliferation of RTE or CD127+CD4+ T cells was determined by Ki67 (B). Th17 cells were determined by the expression of IL-17 and CCR6, Th1 cells by the expression of IFN and CXCR3 (C). Treg cells were defined as FoxP3+. Naturally occurring Treg (nTreg) cells were characterized by CD25 bright expression and CD127-negativity. Furthermore, the proportion of CD4+ T cells expressing IL-10 was determined (D). Supplementary Figure 2. Proportions of inflammatory cytokine secreting effector T cells in differently treated RA patients. Inflammatory cytokine secreting effector T cells in RA patients, treated with csDMARDs-only, TNFi-only or a combination of a TNFi and a csDMARD (methotrexate), compared to HC. csDMARD-only treated patients showed significantly reduced numbers of IFNγg CD4 , IFNγ CD8 (A) or IL-17 CD4 (B) effector T cells, compared to HC. TNFi-only treated patients instead displayed increased numbers of the respective effector T cells. Patients treated with the combination of a TNFi and methotrexate most closely resemble the HC. Bars represent median percentages and interquartile range. Kruskal-Wallis test was performed, followed by Dunn’s test to compare between the study populations. [file 12979_2025_508_MOESM1_ESM.pdf]
