# Supplementary material for: Diverging effects of tumor necrosis factor inhibitors and conventional synthetic disease-modifying antirheumatic drugs on immunosenescence and inflammageing in rheumatoid arthritis: a cross-sectional analysis
Source: Immun Ageing. 2025 May 22;22:21. doi: 10.1186/s12979-025-00508-w (PMC12096643; doi:10.1186/s12979-025-00508-w)
Supplement: Supplementary file 2 — Supplementary Material 2: Supplementary Table 1. Inflammatory and regulatory T cell subsets in inactive and active patients. Supplementary Table 2. Influence of the RA and its pharmacological treatments on the inflammatory T cell subpopulations / the TREC count. Supplementary Table 3. Associations between clinical parameters of the RA and the inflammatory T cell subpopulations / the TREC count. [file 12979_2025_508_MOESM2_ESM.docx]

**Suppl. Table 1**. Inflammatory and regulatory T cell subsets in inactive and active rheumatoid arthritis (RA) patients.

| T cell subpopulation | | HC  (n=20) | Inactive RA  (n=22) | Active RA  (n=16) | p (inactive vs. active RA) |
| --- | --- | --- | --- | --- | --- |
| IFNγ^+^CD4^+^ T cells | | | | | |
|  | Naive | 0.4 (0.2-0.7) | 0.2 (0.2-0.3) | 0.3 (0.1-0.4) | 0.404 |
|  | Effector | 2.0 (0.6-3.2) | 1.5 (0.3-3.6) | 0.5 (0.1-2.4) | 0.341 |
|  | Memory | 6.1 (4.0-9.8) | 3.1 (2.3-5.3) | 4.0 (1.2-4.7) | 0.804 |
|  | TEMRA | 0.2 (0.1-0.5) | 0.2 (0.1-0.7) | 0.3 (0.1-1.1) | 0.919 |
| IFNγ^+^CD8^+^ T cells | | | | | |
|  | Naive | 2.3 (1.7-4.2) | 1.8 (1.3-2.4) | 2.2 (1.3-4.5) | 0.341 |
|  | Effector | 10.1 (4.5-16.8) | 7.5 (2.6-15.9) | 4.6 (1.0-11.4) | 0.284 |
|  | Memory | 7.1 (4.9-11.6) | 5.8 (4.1-8.8) | 6.6 (4.7-9.5) | 0.715 |
|  | TEMRA | 22.4 (12.6-37.9) | 14.8 (6.7-19.0) | 12.0 (5.0-23.5) | 0.849 |
| IL-17^+^CD4^+^ T cells | | | | | |
|  | Naive | 0.12 (0.08-0.21) | 0.10 (0.06-0.16) | 0.10 (0.08-0.16) | 0.759 |
|  | Effector | 0.03 (0.01-0.10) | 0.03 (0.01-0.10) | 0.02 (0.00-0.06) | 0.421 |
|  | Memory | 0.28 (0.19-0.53) | 0.25 (0.14-0.44) | 0.19 (0.13-0.32) | 0.341 |
|  | TEMRA | 0.02 (0.01-0.05) | 0.02 (0.01-0.08) | 0.01 (0.00-0.05) | 0.372 |
| Th1 and Th17 cells | | | | | |
|  | IFNγ^+^CXCR3^+^CD4^+^ T cells | 0.03 (0.01-0.07) | 0.03 (0.02-0.05) | 0.06 (0.03-0.12) | 0.095 |
|  | IL17^+^CCR6^+^CD4^+^ T cells | 0.22 (0.12-0.30) | 0.15 (0.10-0.37) | 0.11 (0.07-0.21) | 0.137 |
| Treg cells | | | | | |
|  | CD25^+^CD127^-^CD4^+^ T cells | 1.8 (0.9-2.7) | 2.2 (1.5-3.4) | 1.7 (1.1-2.5) | 0.201 |
|  | FoxP3^+^CD4^+^ T cells | 0.8 (0.3-1.2) | 1.1 (0.7-1.6) | 0.9 (0.5-1.5) | 0.569 |
|  | IL-10^+^CD4^+^ T cells | 0.5 (0.3-0.6) | 0.4 (0.3-1.0) | 0.5 (0.2-3.9) | 0.965 |

Given is the median (interquartile range) percentage of the respective subset within the CD4^+^ or CD8^+^ T cells. Mann-Whitney U test was performed to compare the T cell subsets in patients with an inactive RA to the subsets in patients with an active RA. The values for the healthy controls (HC) are indicated for comparison.

**Suppl. Table 2**. Influence of the RA and its pharmacological treatments on the inflammatory T cell subpopulations / the TREC count.

| T cell subpopulation | | | RA | TNFi | Methotrexate | Leflunomide | NSAID | Systemic glucocorticoids |
| --- | --- | --- | --- | --- | --- | --- | --- | --- |
| IFNγ^+^ | Effector^#^ | of CD4^+^ T cells | 0,02 | 0,39 | -0,11 | -0,20 | -0,11 | -0,06 |
|  | Memory^##^ |  | **-0,70*** | 0,31 | 0,10 | 0,07 | -0,02 | 0,06 |
|  | TEMRA |  | 0,01 | 0,21 | -0,03 | -0,13 | 0,03 | 0,13 |
| IFNγ^+^ | Effector^#^ | of CD8^+^ T cells | -0,49 | **0,53*** | -0,19 | 0,18 | 0,04 | -0,04 |
|  | Memory |  | -0,31 | 0,07 | -0,03 | 0,27 | 0,15 | -0,10 |
|  | TEMRA |  | -0,45 | 0,19 | 0,04 | -0,03 | -0,01 | -0,13 |
| IL-17^+^ | Effector | of CD4^+^ T cells | -0,14 | 0,39 | -0,14 | -0,09 | -0,16 | 0,00 |
|  | Memory^#^ |  | -0,47 | **0,57*** | -0,10 | 0,39 | -0,24 | -0,01 |
|  | TEMRA^#^ |  | 0,12 | 0,23 | -0,32 | -0,27 | 0,09 | 0,33 |
| Th1 | IFNγ^+^CXCR3^+^ | of CD4^+^ T cells | 0,07 | 0,11 | 0,09 | -0,08 | 0,01 | 0,09 |
| Th17 | IL-17^+^CCR6^+#^ |  | -0,30 | **0,58*** | -0,12 | 0,36 | -0,31 | -0,01 |
| TREC | | | -0,30 | 0,16 | -0,08 | 0,26 | 0,05 | 0,00 |

Depicted are the standardized coefficients of the independent variables of the respective multiple linear regression models. ^#^ indicates a statistically significant regression model (^#^ p<0.05, ^##^p<0.01). * indicates a statistically significant contribution of the respective independent variable to the regression model following correction for multiple testing by applying the two-stage step-up method of Benjamini, Krieger and Yekutieli with a false discovery rate of Q<0.05 (p<0.003). Abbreviations: *TNFi* tumor necrosis factor inhibitor; *NSAID* non-steroidal anti-inflammatory drug. *TEMRA* effector memory T cells re-expressing CD45RA.

**Suppl. Table 3**. Associations between clinical parameters of the RA and the inflammatory T cell subpopulations / the TREC count.

| T cell subpopulation / TREC count | | | TNFi treatment | Sex | Age | Disease duration | Disease activity | RF status | anti-CCP-ab status | ANA status | CRP |
| --- | --- | --- | --- | --- | --- | --- | --- | --- | --- | --- | --- |
| IFNγ^+^ | Effector | of CD4^+^ T cells | 0,55 | -0,04 | 0,02 | 0,15 | 0,00 | 0,06 | -0,15 | -0,12 | 0,22 |
|  | Memory |  | 0,54 | 0,10 | 0,08 | 0,19 | -0,06 | 0,22 | -0,27 | -0,07 | 0,38 |
|  | TEMRA |  | 0,33 | 0,00 | -0,32 | 0,19 | -0,07 | 0,06 | 0,23 | -0,20 | 0,37 |
| IFNγ^+^ | Effector^#^ | of CD8^+^ T cells | **0,86*** | 0,21 | 0,17 | 0,01 | -0,02 | 0,08 | -0,19 | -0,04 | 0,33 |
|  | Memory |  | 0,02 | 0,10 | 0,30 | -0,05 | 0,23 | -0,04 | 0,01 | 0,19 | -0,46 |
|  | TEMRA |  | 0,77 | 0,16 | 0,08 | 0,24 | -0,05 | -0,31 | 0,24 | -0,11 | 0,51 |
| L-17^+^ | Effector | of CD4^+^ T cells | 0,31 | 0,13 | 0,12 | -0,03 | -0,09 | 0,26 | -0,25 | -0,29 | -0,02 |
|  | Memory |  | 0,17 | 0,08 | -0,15 | -0,14 | 0,02 | 0,10 | -0,15 | -0,04 | -0,21 |
|  | TEMRA |  | -0,18 | -0,15 | -0,05 | -0,15 | -0,25 | 0,24 | 0,04 | -0,17 | -0,39 |
| Th1 | IFNγ^+^CXCR3^+^ | of CD4^+^ T cells | 0,07 | -0,14 | -0,06 | -0,01 | **0,60*** | 0,24 | -0,34 | 0,01 | -0,14 |
| Th17 | IL-17^+^CCR6^+^ |  | 0,11 | 0,08 | -0,22 | -0,16 | -0,04 | 0,08 | -0,25 | -0,08 | -0,14 |
| TREC count^#^ | | | 0,07 | -0,03 | **-0,60*** | -0,12 | 0,01 | -0,15 | 0,20 | 0,29 | 0,02 |

Depicted are the standardized coefficients of the independent variables of the respective multiple linear regression models. ^#^ indicates a statistically significant regression model (^#^ p<0.05). * indicates a statistically significant contribution of the respective independent variable to the regression model following correction for multiple testing by applying the two-stage step-up method of Benjamini, Krieger and Yekutieli with a false discovery rate of Q<0.05 (p<0.006).
